# Management of anterior chamber dislocation of a dexamethasone intravitreal implant: a case report

**DOI:** 10.1186/s13256-016-1077-2

**Published:** 2016-10-13

**Authors:** Fernanda Pacella, Enzo Agostinelli, Sandra Cinzia Carlesimo, Marcella Nebbioso, Roberto Secondi, Michele Forastiere, Elena Pacella

**Affiliations:** 1 Department of Sense Organs, Faculty of Medicine and Dentistry, Sapienza University of Rome, Viale del Policlinico, 00161 Rome, Italy; 2 Department of Biochemical Sciences, Sapienza University of Rome, Piazzale Aldo Moro 5, 00185 Rome, Italy

**Keywords:** Ozurdex, Ozurdex dislocation, Intravitreal dexamethasone implant, Cystoid macular edema, Case report

## Abstract

**Background:**

Ozurdex is a 700 mcg dexamethasone intravitreal implant, approved for the management of macular edema secondary to retinal vein occlusion, and other related pathoglogiesAnterior chamber dislocation of Ozurdex represents an uncommon complication of the intravitreal injection, which can be managed by repositioning the implant into the vitreous cavity. We describe the case of a successful repositioning of an Ozurdex implant by mobilization and subsequent balanced saline solution injection in the anterior chamber.

**Case presentation:**

An 83-year-old white woman presented to our Emergency Unit complaining of pain and vision loss in herright eye lasting a week. Her anamnesis revealed a history of persistent cystoid macular edema after phacoemulsification with scleral-fixated posterior chamber intraocular lens implantation, recently treated with an intravitreal Ozurdex implant. She also took a long-distance flight 2 days after the injection.

An anterior segment examination showed corneal edema and the rod implant adherent to corneal endothelium. To avoid corneal decompensation, we opted for a implant repositioning. Under topical anesthesia, a 30-gauge needle was introduced through a limbar incisionto mobilize the dislocated rod. Balanced saline solution was injected, with a successful repositioning of the implant into the vitreous cavity. Topical 5 % hypertonic saline solution and 0.2 % betamethasone associated with 0.5 % chloramphenicol drops were administered four times a day. To prevent redislocation of the Ozurdex implant, she was instructed to avoid prone position, any kind of physical effort, and not to undertake long-distance flights during the first postoperative week. One week after surgery, an anterior segment examination showed an improvement of corneal edema. Funduscopy showed that the Ozurdex implant was settled into the vitreous cavity.

**Conclusions:**

Anterior chamber dislocation of Ozurdex from the vitreous cavity is rare. In our patient, in addition to the posterior capsule tearing, the long-distance flight could have contributed to implant dislocation. Repositioning of the implant is necessary to avoid endothelial decompensation. It can be carried out by using saline balanced solution with the same efficacy as other surgical procedures reported in the literature. A possible disadvantage of this procedure could be implant migration.

## Background

Ozurdex (Allergan Inc., Irvine, CA, USA) is a dexamethasone (700 mcg) rod-shaped biodegradable implant of 6 mm in length and 0.46 mm in diameter, which is injected by a 22-gauge needle into the vitreous cavity. The Ozurdex (dexamethasone) intravitreal implant has been approved to manage several ocular diseases including macular edema due to retinal vein occlusion [1, 2], noninfectious uveitis affecting the posterior segment [3, 4], and diabetic macular edema [5]. The efficacy and safety of the Ozurdex (dexamethasone) implant has been reported in the literature, including cases of Irvine-Gass syndrome [6].

Despite this evidence, rare complications associated to the Ozurdex (dexamethasone) implant have been described. These include not only long-term adverse events in cases of required repeated dexamethasone 0.7 mg injections, such as cataract development and secondary glaucoma [7], but also complications due to the implant itself, such as desegmentation (fracture) of the implant [8], accidental injection of Ozurdex (dexamethasone) into the crystalline lens [9], and migration of the Ozurdex (dexamethasone) implant into the anterior chamber [10].

Anterior chamber dislocation of an Ozurdex (dexamethasone) implant represents an uncommon complication of this procedure, which can be managed by extracting the implant from a sclerocorneal incision or repositioning to the vitreous cavity [10, 11]. We describe the case of an efficient repositioning of an Ozurdex (dexamethasone) implant by mobilization and subsequent balanced saline solution injection in the anterior chamber.

## Case presentation

An 83-year-old white woman presented to our Emergency Unit with pain and vision loss in her right eye for the past week. Her anamnesis revealed a history of persistent cystoid macular edema after phacoemulsification with scleral-fixated posterior chamber intraocular lens implantation in her right eye, previously managed by an intravitreal injection of Ozurdex (dexamethasone); in addition, her left eye was operated on due to cataracts without complications. She also informed us of a long-distance flight she took from Italy to Australia 2 days after the intravitreal injection.

**Fig. 1:**
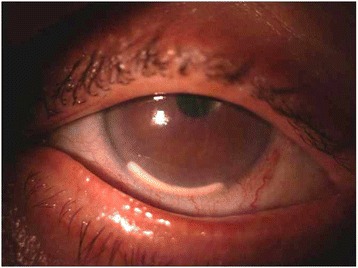
Slit-lamp biomicroscopy showing the dexamethasone implant dislocated in the anterior chamber. The implant is located just in front of the iris, in the lower part of the visual axis

Anterior segment examination of her right eye showed conjunctival injection, corneal edema, and the rod implant dislocated in the anterior chamber, adherent to corneal endothelium (Fig. 1). The intraocular pressure during the first visit measured 11 mmHg. After the repositioning of the dexamethasone implant and throughout her follow-up visit, her intraocular pressure never exceeded 17 mmHg in our case study. The dexamethasone implant not only decreased her macular edema but also helped to improve her visual acuity. Her best spectacle corrected visual acuity (BSCVA) was 1.69 LogMAR.

After evaluation of her clinical condition and of the high risk of corneal endothelial decompensation due to the toxic action of the rod, we decided to remove it from the anterior chamber. The surgery was performed at our Department of Sense Organs. Informed consent was obtained from our patient. The study adhered to the tenets of the Declaration of Helsinki for research involving humans.

Clinical intervention was performed under sterile conditions in the operating room. The repositioning of the intravitreal dexamethasone implant was performed under sterile conditions and after preparation of her conjunctiva using 5 % povidone-iodine solution. Under topical anesthesia with ropivacaine and positioning of the blepharostat, a limbar incision was made and a viscoelastic substance was inserted into the anterior chamber to avoid damaging adjacent structures. A 30-gauge needle was introduced to mobilize the dislocated rod. Then, balanced saline solution was injected and a successful repositioning of the implant in the vitreous cavity was obtained. The viscoelastic substance was removed from the anterior chamber.

After the repositioning of the dexamethasone implant, our patient was administered an eye drop formula consisting of 5 % hypertonic solution to reduce the edema of her cornea for 3 months, 3 times per day. This was combined with 0.2 % betamethasone associated with a 0.5 % chloramphenicol drop therapy that was administered in descending doses: in the first week four drops per day, second week she administered three drops per day, the third week the dose consisted of two drops a day, and later it was tapered during her postoperative period.

**Fig. 2:**
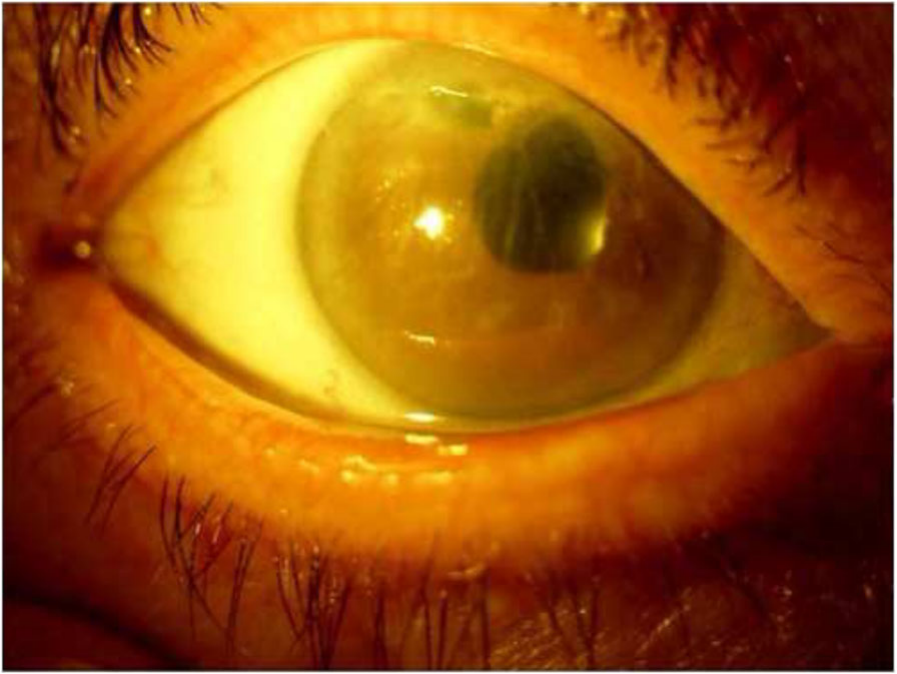
Slit-lamp biomicroscopy showing the result of repositioning of the dexamethasone implant. Corneal edema

One week after surgery, she did not complain of more pain and reported an improvement in visual acuity. Her BSCVA was 1 LogMAR. A fundus examination showed that the Ozurdex (dexamethasone) implant was settled in the vitreous cavity (Fig. 2).

A clinical biomicroscopy examination showed absence of inflammatory reaction, partial improvement of corneal edema, and the anterior chamber optically empty. Ethics approval for the procedure described was obtained from our local committee.

## Discussion

Anterior chamber dislocation of an Ozurdex (dexamethasone) implant from the vitreous cavity is a rare complication which occurs more frequently in aphakic eyes or in cases of tear of lens capsule and prior vitrectomy [10]. In addition, unusual body postures, such as face down or prone positions, can also be a risk factor for anterior dislocation [12]. To prevent dislocation, the patients must be instructed not only to avoid a prone position or physical efforts, but also not to undertake long-distance flights during the first weeks after Ozurdex (dexamethasone) intravitreal implant. Once the airplane has taken off, to ensure the safety of passengers within the cabin, air pressure is reduced. Due to the decreased air pressure within the cabin, our internal pressure can rise within our body cavities causing major complications in people who have had eye surgery, such as our patient. The difference in air pressure increased the pressure of the vitreous with the dexamethasone implant dislocation in the anterior chamber [13]. In the present case report, the sudden pressure changes during the long-distance flight could have contributed to the dislocation of the implant.

Anterior chamber dislocation of Ozurdex (dexamethasone) intravitreal implant can be managed by extracting the implant from a sclerocorneal incision or repositioning to the vitreous cavity [6]. Repositioning of the rod can be managed by different strategies. Although cases of spontaneous relocation of the implant in the vitreous cavity have been reported [14], repositioning of the implant is necessary to avoid irreversible corneal decompensation. Here we describe the case of an efficient repositioning of an Ozurdex (dexamethasone) implant by mobilization and subsequent balanced saline solution injection in the anterior chamber.

The present case report suggests that the efficacy of mechanical repositioning, carried out using saline balanced solution, can be similar to the efficacy of other surgical procedures reported in the literature. Mechanical repositioning has the advantage of minimizing corneal toxicity and avoids the use of more invasive techniques. The migration of the dexamethasone implant in the anterior chamber took place 2 days after the intravitreal injection. The migration persisted for a total of 10 days, including 2 days necessary for patient’s preparation to the surgical repositioning of the implant into the vitreous cavity.

The dexamethasone implant was inserted in her eye in Italy, before going to Australia for vacation. During her time in Australia complications arose and it was necessary for her to visit a hospital. The medical staff in Australia informed her of the seriousness of the problem and requested surgery. She opted to return to Italy for her surgery as she felt more comfortable with Italian physicians.

This study is in line with other studies showing that mechanical repositioning may reduce the need for surgical intervention. It has been shown that repositioning of the implant can be achieved with the use of a 30-gauge needle under topical anesthesia at the slit lamp [15] or with the patient posturing to reposition the dexamethasone implant back into the vitreous cavity [16–18]. Despite this evidence, the choice of the technique adopted should depend on the location and positioning of the dexamethasone implant relative to other intraocular structures. Thus, surgical procedures are sometimes inevitable to relocate the implant and to manage corneal decompensation and elevated intraocular pressure [19, 20].

## Conclusions

In conclusion, this case report indicates that implant repositioning can be successfully achieved by mobilization and subsequent balanced saline solution injection in the anterior chamber. The procedure is minimally invasive and, due to its celerity, it can have beneficial effects on corneal edema caused by an Ozurdex (dexamethasone) rod in contact with endothelium. However, the disadvantage of this procedure can be the risk for implant migration. Patients must be instructed not only to avoid a prone position or physical effort, but also not to undertake long-distance flights during the first weeks after Ozurdex (dexamethasone) intravitreal implant.

## Abbreviations

BSCVA: Best spectacle-corrected visual acuity
